# Quality of Nursing Care: Addressing Sexuality as Part of Prostate Cancer Management, an Umbrella Review

**DOI:** 10.1111/jan.16703

**Published:** 2025-01-09

**Authors:** Catherine Neenan, Anna V. Chatzi

**Affiliations:** ^1^ Department of Nursing and Midwifery University of Limerick Limerick Ireland

**Keywords:** barriers, nursing, prostate cancer, quality care, sexuality

## Abstract

**Background:**

Sexuality is a fundamental aspect of health and wellbeing. The management of prostate cancer can result in erectile dysfunction and body feminisation, resulting in loss of masculinity and alterations of body image. Prostate cancer patients identify sexuality as an unmet need and report little or no communication with their healthcare providers on the topic.

**Aim:**

This umbrella review aims to determine the barriers that may preclude nurses from discussing sexuality with prostate cancer patients.

**Design:**

An umbrella review of systematic review studies was undertaken using the PRISMA guidelines.

**Method:**

Five databases were comprehensively searched, CINAHL, MEDLINE, PsycINFO, Cochrane and Prospero, from October 1, 2013 to December 1, 2023, using the defined criteria. A total of 11 systematic reviews were included in this review consisting of 10 with quantitative and 1 with quantitative/mixed methods approach.

**Results:**

This study identified common themes, which were categorised into four groups: (a) lack of training and education, (b) age and years of clinical experience of nurses, (c) personal values and attitudes and (d) organisational factors. These factors contribute to why nurses feel unprepared and admit to not having adequate knowledge or expertise to have this discussion.

**Conclusion:**

The findings of this study illustrate that nurses require specialised communication skills to manage sensitive discussion with patients. Education is crucial to facilitate and empower nurses to discuss sexuality with their patients. Developing a pathway to specialist referrals will encourage nurses to address this with their patients.

**No Patient or Public Contribution:**

For the preparation of this paper, no direct involvement of patients or public has been deemed applicable to this work. This is an umbrella review paper.

**Summary**

- Prostate cancer patients identify sexuality as an unmet need and report little or no communication with their healthcare providers on the topic.
- The identification and understanding of why communication on the topic of sexuality does not take place are done. This investigation is a leading research to determine the variables or barriers that exist and preclude nurses from addressing sexuality with prostate cancer patients.
- Addressing the sexuality of prostate cancer patients will greatly improve patients' quality of life and facilitate a significantly improved cancer journey. This could be possible by:
  ○ Integrating mandatory education on sexuality in specialised nursing programs will help nurses navigate any taboos or discomfort associated with this subject.
  ○ Establishing a comprehensive referral system to experts in the field of sexual health is also essential.
  ○ A collaborative approach involving various healthcare professionals can create a supportive environment for all.

## 1 Introduction

A vital component of every human being's quality of life is sexuality (Bolin et al. 2021; Carter et al. 2018; Nery‐Hurwit et al. 2022). The World Health Organisation (2002) defines sexuality as ‘a central aspect of being human throughout life and encompasses sex, gender identities and roles, sexual orientation, eroticism, pleasure, intimacy and reproduction’ (World Health Organisation 2002, 10). All of these elements are affected when patients are faced with a serious health event such as a diagnosis of cancer.

### 1.1 Background to This Review

A patient's sexual health can be severely impacted by the outcomes of receiving a cancer diagnosis and undergoing therapy (Onukwugha et al. 2017; Walker et al. 2021), which can have a profound effect on a patient's personal and sexual world (Moore, Higgins, and Sharek 2013). The interconnecting factors of masculinity, body image and self‐esteem have a significant impact on the quality of life for prostate cancer patients (Bowie et al. 2022). It is evident that prostate cancer is one illness that highlights the detrimental effects the treatment can have on sexuality (Chambers et al. 2017; Salifu, Almack, and Caswell 2023). The range of available treatment options can result in incontinence, infertility, feminisation and persistent alterations to sexual function including erectile dysfunction, penile atrophy and loss of libido (Donovan et al. 2018; Lynch, O'Donovan, and Murphy 2019). The estimated prevalence of erectile dysfunction is between 70% and 90% (Fode and Sonksen 2014).

Discussion on sexuality with patients remains sparse, requiring improvements in the communication of issues with sexuality between patients and health care professionals (Ussher et al. 2013). Saunamaki, Andersson, and Engstrom (2010), when carrying out a survey of nurses in Sweden, discovered that while nurses appreciate the significant effect illness and treatment can have on patients' sexuality, 80% of them did not allocate time to address sexual concerns. This alarming fact is reiterated by Sporn et al. (2015), who identified that 90% of cancer survivors reported that healthcare professionals infrequently asked them about their sexuality. This inaction and gap in the supportive aspect of holistic care is fundamental to why patients are reporting their unmet needs in their supportive care (O'Brien et al. 2011; Paterson et al. 2015; Wasserug, Westle, and Dowsett 2017).

### 1.2 Prostate Cancer

Globally, prostate cancer is the second most prevalent type of cancer (Wang et al. 2022) with approximately 1.3 million men diagnosed worldwide in 2018 alone (Bray et al. 2018). In Ireland, prostate cancer is the most common cancer among men, excluding skin cancer, with over 4000 men diagnosed in 2021 (National Cancer Registry Ireland 2022). For example, using a demographic projection, it is estimated that the number of prostate cancer diagnosis in Ireland will increase from 3214 in 2015 to 6869 in 2045, an increase of 114% (National Cancer Registry Ireland 2019).

Despite the high survival rates of prostate cancer (Allemani et al. 2015; Maharaj et al. 2021), both the diagnosis and treatments for prostate cancer can negatively affect a person's quality of life (Punnan et al. 2015; Watson et al. 2016). Research has revealed that, among men diagnosed with prostate cancer, sexual dysfunction is the most common health‐related complaint (Chambers et al. 2017; Tsang et al. 2018).

Treatment for prostate cancer includes prostatectomy, radiotherapy androgen deprivation therapy and chemotherapy (Department of Health 2015). All of these treatments can potentially affect sexual function and sexuality (Carter et al. 2018; Chambers et al. 2017; Eroglu and Ozkan 2023). Side effects commonly reported from these treatments include erectile dysfunction, diminished libido, pain, fatigue, low mood, altered body image, shortening of the penis, incontinence, body feminisation and disrupted masculinity (Carter et al. 2018; Chambers et al. 2017; Graugaard 2017; Walker et al. 2021; Watson et al. 2016). Depression and anxiety are considerably higher in men with prostate cancer when compared with the general population (Watts et al. 2014), as is the risk of suicide (Klaassen et al. 2015; Matheson et al. 2020; Sporn et al. 2015). While awareness is observed among oncology nurses that sexuality is a vital component of holistic care, there is a wealth of evidence highlighting that nurses avoid the topic and neglect to communicate about sexuality with patients (Macleod and Nhamo‐Mhuire 2016; O'Connor, Drummond, et al. 2019; Reese et al. 2017; Winterling, Lampic, and Wettergren 2020). At the same time, prostate cancer patients desire open communication about sexuality (Katz and Dizon 2016), as many patients report low rates of communication on the topic (Carter et al. 2018; O'Connor, Connaghan, et al. 2019; Sporn et al. 2015). This has been identified as factors for lack of holistic care, limiting patients' quality of life and their survivorship journey (Paterson et al. 2017; Saunamaki and Engstrom 2014; Watson et al. 2016).

The Irish National Cancer Strategy (Department of Health 2017) strives to address patients' needs and aims to enhance services for cancer survivors and address their unmet needs. While efforts have been made, particularly relating to sexuality of cancer patients (Department of Health 2017), there is a recognised need for further work in this direction. The Code of Professional Conduct and Ethics states that the nurse must provide the highest standard of quality care that is evidence‐based to each patient (Nursing and Midwifery Board of Ireland 2021). It is essential that nurses are prepared to facilitate a discussion on sexuality with their prostate cancer patients. This umbrella review aims to create evidence‐based research that identifies the factors that influence discussion on sexuality with prostate cancer patients and the barriers that impede them. This study aims to highlight opportunities that may address this unmet need which may subsequently improve the quality of life for prostate cancer patients.

The research questions of this umbrella review were:

1. What are the factors that affect the discussion of sexuality between nurses and prostate cancer patients?
2. How do nurses address sexuality when caring for patients with prostate cancer?

## 2 Methodology

### 2.1 Eligibility Criteria and Information Source

The acronym PICOS (Participants, Intervention, Comparison, Outcomes and Studies) (Akers 2009) was used to address the study's research questions (Table 1). Prostate cancer patients and nurses and healthcare professionals were included in the participants section, while the discussion of sexuality between participants was the core aim. The searched intervention included the factors that affect this discussion along with the strategies used to enable this discussion and the comparison was towards nurses' attitudes, patients' views and any other identified variables that were highlighted by the examined studies.

**TABLE 1:** PICOS.

Participants	Prostate Cancer Patients Nurses and Health Care Professionals
Intervention	Factors that affect the discussion of sexuality between nurses and prostate cancer patients Strategies used to help address the topic of sexuality with prostate cancer patients
Comparison	Studies identifying variables that affect the discussion on sexuality between nurses and prostate cancer patients Studies reporting nurses' attitudes on sexuality Studies identifying patients view on nurses addressing sexuality
Outcomes	Identification of factors in addressing prostate cancer patients' sexuality and strategies used by nurses to address sexuality
Studies	Systematic review studies

Databases were searched from 1st October 2013 to 1st December 2023. The last day of the search was 15th December 2023.

To meet the inclusion criteria (Table 2), articles were obtained from peer‐reviewed journals, within the past 10 years, published and fully translated in English. Within the umbrella review requirements, only review papers were included with population of nurses and healthcare professionals dealing with the topic of male and lesbian, gay, bisexual and transgender (LGBTQ) sexuality with prostate cancer patients. In an effort to obtain a wide scope of cultural backgrounds, international studies were included, but only those translated to English. Studies focusing on student nurses only were excluded. Complete information regarding inclusion and exclusion criteria can be found in Table 2.

**TABLE 2:** Eligibility criteria.

Selection criteria	Inclusion criteria	Exclusion criteria
Language	English or fully translated into English	Non‐English
Dates	Publications from Jan 2013 to Dec 2023	Publications before 2013 Publications after 2023
Study type	Systematic review Cross‐sectional Reviews	Quantitative research Qualitative research Mixed methods research Editorials News articles Thesis or dissertations Grey literature
Population	Nurses aged 18 years and older Graduate and undergraduate nurses Healthcare professionals including nurses, nurse managers, nurse practitioners and clinical nurse managers Prostate cancer patients, chronic illness including prostate cancer and older population including prostate cancer	Nurses aged under 18 years Undergraduate nurses only Physicians, surgeons, social workers and physiotherapist alone Patients with cancer diagnosis excluding prostate cancer
Topic	Male sexuality Sexual health education Lesbian, gay, bisexual and transgender (LGBTQ)	Domestic violence Sexual abuse Rape

### 2.2 Study Selection

**FIGURE 1:**
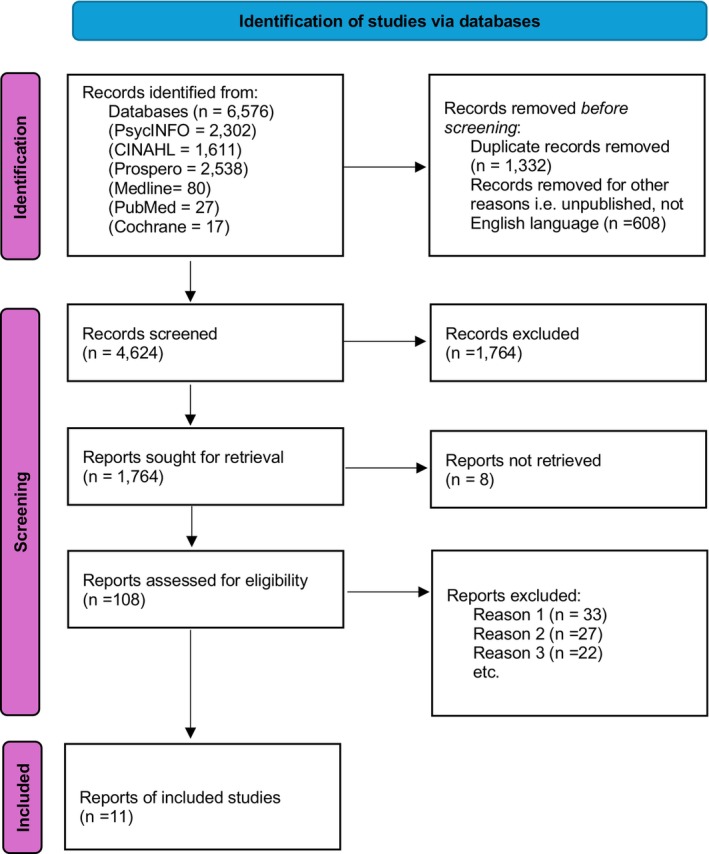
PRISMA flow diagram.

This review was conducted in accordance with the Preferred Reporting Items for Systematic Reviews (PRISMA; Page et al. 2021; Figure 1). A systematic search was carried out comprising of search terms grouped in the following areas: (a) prostate cancer, (b) nursing, (c) sexuality and (d) barriers to communication. The search included a combination of Boolean operators, truncation markers and MeSH headings along with keywords, phrases and synonyms including prostate neoplasm, sexuality, sexual health, attitudes to sexuality, effective communication, healthcare professionals and nurses, which was were used in the search to enhance the searches' sensitivity and inclusivity. A search of five electronic databases (CINAHL, Medline, PsycINFO, Cochrane and Prospero) was conducted with the assistance of a research librarian. Two search runs proceeded the final search run. The two initial search runs were conducted within the same five electronic databases and served the purpose of the search terms refinements. Reference lists of included articles were also examined.

### 2.3 Data Collection

The quality of each review article was assessed by the JBI Critical Appraisal Checklist for Systematic Reviews and Research Syntheses (Joanna Briggs Institute 2017). Titles and abstracts of the discovered articles were firstly assessed according to inclusion criteria by a sole investigator (CN), while a second investigator (AC) was available to resolve any issues on individual items. Then the two investigators independently examined the articles' full texts. Discussion and consensus were the process that was followed in the decision of the finally selected articles. Titles and abstracts of selected articles were then stored in the Endnote database.

Data from each of the 11 articles that were kept for this study were then extracted into an Excel spreadsheet containing information on authors, country, study aim, study setting, sample, data collection method and main findings. Extracted data is presented in Table 3.

**TABLE 3:** Data extraction table.

No	Author, year and country	Study design	Aim of study	Study setting	Sample	Data collection method	Main findings	Quality
	Aling et al. (2021), Sweden	Quantitative	Aimed to map, synthesise, findings regarding barriers and enabling factors for nurse–patient SSH discussions	A scoping review inspired by Arksey and O'Malley	19 articles published between 2009 and 2019	Cinahl and Medlars were databases used	Barriers and enablers identified. Enabling factors: Core values and availability of resources and Barriers: beliefs and attitudes, fear of convictions, and work‐related factors	Good
2.	Bauer, Haesler, and Fetherstonhaugh (2016), Australia	Quantitative	To report on the findings of a systematic review, which examined the experiences and views of older people on health professionals' recognition of sexuality and sexual health and whether these aspects of the person are incorporated into care	A systematic review of literature identified from 11 platforms identifying the topic of sexuality and older patients were investigated	18 heterogeneous studies were reviewed, 7 quantitative, 8 qualitative and 3 opinion papers	43 findings were extracted and grouped into 14 categories. 5 syntheses summarised both quantitative and qualitative evidence and	Sexuality is important for many older people The topic is not commonly addressed by health care providers due to embarrassment, dissatisfaction with treatment, negative attitudes and seeming disinterest	Good
3.	Chen, Jones, and Bannatyne (2020), Australia	Quantitative Systematic review	To establish an understanding of health care professionals' knowledge and attitudes and to identify the tools used to assess sexuality in older people and those identified as lesbian, gay, bisexual, trans or intersex (LGBTI)	Studies reporting on qualitative and/or qualitative research on knowledge and/or attitudes related to sexuality in older people and LGBTI individuals and of study participants who were healthcare professionals	A total of 19 articles within a 10 year period was deemed eligible for inclusion in this review	Integrative approach to provide a comprehensive overview in relation to the phenomenon of interest	(1) HCPs levels of knowledge and attitudes can depend on their professional position, qualification, personal beliefs and years of work experience (2) There is a critical need for professional development learning opportunities and care guidance (3) There are limited well‐validated tools to assess knowledge and attitudes	Good
4.	Fabrício et al. (2021), Brazil	Quantitative Literature Review	Identify health care professionals approach to sexuality in the elderly	Integrative review of published research within a 10 year period reviewing healthcare professionals approach to sexuality and the elderly	14 international articles consisted of between 52 and 112 health professionals, mostly nurses, who cared for patients in acute and long‐term care facilities	Integrative review of 14 carefully selected research articles published in peer‐reviewed journals, focusing on healthcare professionals and sexuality in elderly patients	(1) Most healthcare professionals have limited knowledge, varied attitudes on sexuality and do not approach the topic proactively (2) The lack of knowledge and training on sexuality is a deterrent for staff from discussing the topic (3) Lack of time in appointments and discomfort contributed to the difficulties	Fair
5.	Fennell and Grant (2019), USA	Quantitative	Explore current and relevant evidence regarding factors that influence nurses' provision of sexual healthcare education to patients	Articles from peer‐reviewed journals on the topic of communication and sexual health education for patients	10 articles, qualitative, quantitative and mixed methods from 2012 to 2017 focused on nursing staff	4 categories for collection of data: Knowledge, attitudes and beliefs, nurses' comfort and perceived barriers	Sexual healthcare is not being discussed Knowledge, attitudes and beliefs Nurses' comfort, and perceived barriers need to be addressed at the educational level, workplace level and professional society	Good
6.	Haesler, Bauer, and Fetherstonehaugh (2016), Australia	Systematic Review	Identify the knowledge and attitudes of health professionals towards sexuality and sexual health of older patients	23 research articles were deemed eligible. These consisted of both qualitative (8) and quantitative (15) articles	14 cross sectional surveys were included and 1 used as an observational pre/post test design. Qualitative studies used grounded and ethnography identifying themes of ‘Knowledge of sexuality and sexual health of older people’ and ‘Attitudes towards sexuality and sexual health of older people’	Quantitative and Qualitative Research was obtained which offered unique commentary. Papers were then critically appraised by 2 independent reviewers using the Joanna Briggs Institute extraction tools	Healthcare staff do not regard sexuality as significantly important for older patients and believe older patients do not wish to discuss the topic of sexuality Healthcare can have a medicalised viewpoint of sexuality and the attitudes of healthcare regarding sexuality and older patient is influenced by society, and personal viewpoints can influence how healthcare view sexuality and older patient	Good
7.	O'Connor et al. (2019), United Kingdom	Quantitative	To explore healthcare professional‐perceived barriers and facilitators to discussing sexual health and wellbeing with patients diagnosed with chronic illness	A systematic review of qualitative and quantitative literature over a 17 year period	30 research articles, 30 qualitative and 18 quantitative studies were used to examine perspectives of healthcare professionals discussing sexual wellbeing with patients diagnosed with chronic illness and explore factors perceived to be barriers of facilitators to this discussion	52 concepts were reciprocally translated into 5 themes: (1) individual and societal attitudes to sex and sexual wellbeing (*n* = 13), (2) patient‐specific factors (*n* = 14), (3) organisational and professional factors (*n* = 17), (4) strategies to overcome barriers (*n* = 4) and (5) perceived training needed (*n* = 4)	Inter‐related factors significantly influence perspectives of healthcare professionals on discussing sexual wellbeing with patients. Staff discomfort and fear of embarrassment strongly influence staff initiating discussions Further education is required for staff on this topic Organisational changes to allow privacy for discussions, staff shortages and privacy will be required to enhance staff‐patient discussions on sexual wellbeing	Good
8.	Papadopoulou et al. (2019), Scotland	Quantitative	To establish the state of knowledge pertinent to nurses' competencies in delivering sexual healthcare to patients with cancer, to better understand moderating factors and evaluate interventions to enhance nurses' competencies	A systematic review of quantitative, qualitative and mixed methods were studied	31 articles were studied, 21 quantitative, 7 qualitative and 3 mixed methods studies were eligible following rigourous searches of 9 platforms between 2008 and 2018	4 research questions were underpinned in this systematic review: (1) what are nurses' perceived competencies in providing sexual health care to oncology patients (2) What are the barriers and facilitators? (3) Professional development and (4) are these interventions effective?	Nurse‐led provision of sexual health in oncology is sub‐optimal. Nurses' assumptions and prejudice, lack of confidence and challenges of the infrastructure of the health system	Good
9.	Reese et al. (2017), USA	Quantitative Mixed Methods	To examine the prevalence of and factors associated with patient–provider communication about sexual concerns and cancer	A systematic review of empirical studies on the topic of communication between health care professionals and oncology patients regarding sexual concerns	Search of 29 studies from 10 countries including USA, Netherlands, Canada and Australia between 2000 and 2015	Communication by NCCN guidelines was the framework used	Sexual issues go unaddressed. More discussions on sexual health with men Nurses with more experience discuss the topic	Good
10.	Vassao et al. (2018), Brazil	Quantitative	To identify intervening factors in the approach to sexuality by patients and professionals and to describe strategies used in this approach	Studies from years 2007 to 2017 in Portuguese, English and Spanish concerning factors in the approach to sexuality by healthcare professionals and cancer patients	An intregrative literature review of 18 articles, both qualitative and quantitative articles	2 categories identified, (1) patients reasons for not discussing sexuality and (2) healthcare professional reasons	Sexuality is overlooked. Barriers including lack of education and expertise, lack of dialogue and opportunity, focus on the cancer disease and lack of standards to guide the conversation Strategies to address sexuality include onsite programs, printed leaflets, and active methodology for small groups	Good
11.	Williams and Addis (2021), England	Quantitative	To ascertain the educational requirements of health professionals in oncology and palliative care to enable effective assessment and support to patients	Literature review of articles pertaining to the topic of addressing patient sexuality issues in cancer and palliative care	Review qualitative (6) and quantitative literature (7), also mixed methods (5) and evaluation reports (2) from 5 electronic databases	Not outlined	Discovery of numerous barriers preventing professionals from discussing sexuality: (1) lack of knowledge, (2) poor confidence, (3) limited time, (4) poor environment, (5) assumptions from staff about patients and their sexuality	Fair

### 2.4 Data Synthesis

Studies were assessed using the 10‐item Critical Appraisal Skills Programme (CASP). This checklist identifies the clear aims of the research, while also assessing if appropriate methodology was used. It takes a close look at the sample strategy and data collection process, while also investigating the relationship between the researcher and its participants and identifying any ethical issues. By addressing the data analysis, this appraisal checklist verifies the research findings and the value of the research by highlighting if the results can be adapted to other populations (Long, French, and Brooks 2020). This tool has three answer options for each of the 10 questions (‘Yes = 1’, ‘Can't Tell = 0’ and ‘No = 0’), which are then summed giving a score of 0–10. A score of ≤ 3 is graded as ‘Poor’, 4–6 ‘Fair’ and > 7 ‘Good’ (Table 3). Any incongruity was resolved with the second investigator (AC).

## 3 Results

A total of 6676 records were obtained from database searches which were exported to Endnote and screened. With the removal of duplicates, 4234 remained. Once abstracts and titles were received, these papers were further classified based on the study's inclusion and exclusion criteria of published articles in the English language within the specified time. 108 records were selected for a full text review. Additional papers were identified through hand searches of relevant journals. This resulted in 11 papers that met the inclusion criteria (10 systematic reviews and 1 scoping review that included synthesis of the examined papers). These 11 papers, being a systematics review, have been included in total 271 identified various (research, reviews, etc.) papers. Figure 1 shows the number of papers identified, screened and included in the final umbrella review.

Of the 11 papers identified, 10 were quantitative and 1 quantitative/mixed methods in their approach. Table 1 outlines the main elements of the examined studies. The studies were carried out internationally, including research from Sweden (1), United States of America (2), Australia (2), United Kingdom and Scotland (3) and Brazil (2).

Studies included various types of healthcare professionals such as nurses, doctors, physiotherapists and social workers. These professionals have been providing care to patients with prostate cancer or chronic illness, including prostate cancer. Especially for nurses, all articles in this study reveal their appreciation to the fact that a patient's sexuality is significantly impacted, following a diagnosis of prostate cancer and the available treatments. Despite this, there is overwhelming evidence identifying barriers that hinder the discussion of sexuality with patients (Aling et al. 2021; Fennell and Grant 2019).

All included papers come from various countries around the world representing Western, Eastern and other cultures, mainly Western countries, and can be found in Table 4.

**TABLE 4:** Countries of the papers included in this paper.

Countries	Number of papers
Sweden	1
Australia	3
Brazil	2
USA	2
UK	3

*Note:* Blue = Western cultures, Orange = Eastern cultures, Gree = other cultures.

The origin of these papers might provide insight on the geographical areas in which the enquiry of this topic is a priority and indicative of the relevant research activity there. However, these papers are systematic reviews themselves, and the location of the authors does not reflect the actual relevant research activity. To get further insight, the origin of the included studies in each one of the 11 review papers, as they were reported in these review papers, are presented in Table 5. Table 5 shows that studies from multiple cultures have been included. In this table, it is shown that Western cultures are more focused on this topic, followed by Eastern and other cultures.

**TABLE 5:** Countries of all papers reviewed by the papers included in this study.

Country	Mentioned in papers with numerical value‐total	Additional studies mentioned in papers without numerical value
Netherlands	17	Yes
Sweden	8	Yes
UK	9	Yes
Turkey	7	Yes
USA	27	Yes
Canada	7	Yes
Australia	18	Yes
New Zealand	3	
Thailand	1	
China	13	Yes
Zimbabwe	1	
Taiwan	3	
Ireland	9	Yes
Brazil	4	Yes
Belgium	2	Yes
Iceland	2	Yes
S. Africa	1	
Finland	1	
Norway	1	
Egypt	Yes	Yes
S. Korea	Yes	Yes
Morocco	1	
Malaysia	1	

*Note:* Blue = Western cultures, Orange = Eastern cultures, Green = other cultures.

A narrative synthesis of the data was conducted, structured around (a) the barriers that preclude nurses from discussing sexuality with prostate cancer patients and (b) acknowledging sexuality as an unmet need for these patients.

Emerging themes identified in this research were categorised into four groups: (a) lack of training and education, (b) age and clinical years of experience of nurses, (c) personal values and attitudes and (d) organisational factors. These themes help identify the barriers that hinder nurses discussing sexuality with prostate cancer patients and also provide insight into why patients are experiencing an unmet need.

### 3.1 Lack of Training and Education

Training and education have been revealed to be a highly discussed theme among the examined studies. A notable 55% of the studies with nursing participation identified insufficient training and education as the primary obstacle to discussing sexuality with prostate cancer patients (Bauer et al. 2013; Fabrício et al. 2021; Fennell and Grant 2019; O'Connor, Connaghan, et al. 2019; Vassao et al. 2018; Williams and Addis 2021). Evidence shows that nurses with additional training are more confident in initiating conversations about sexuality (Reese et al. 2017). As training and education are activities that need motivation and effort, the openness of nurses in attending such programmes was also discussed and revealed in the need for enhanced relevant education (Chen, Chang, et al. 2020; Fennell and Grant 2019; O'Connor, Connaghan, et al. 2019; Papadopoulou et al. 2019; Reese et al. 2017; Williams and Addis 2021).

In an effort to enable this education process for nurses, various educational methods are proposed, including online courses and interactive workshops (Papadopoulou et al. 2019). Papadopoulou et al. (2019) suggested a two‐tiered approach to improve communication skills and promote discussion of sexuality across all nursing levels. Integrating sexuality to formal nurse training could clarify and detangle its distinction from sexual health (Fennell and Grant 2019).

### 3.2 Age and Clinical Years of Experience

Data obtained from these studies showed nurse's age and clinical experience to impact their comfort level in discussing sexuality with prostate cancer patients (Chen, Chang, et al. 2020; Fennell and Grant 2019). In particular, Chen, Chang, et al. (2020) and Chen, Jones, and Bannatyne (2020) found that older nurses with extensive clinical experience are more at ease discussing sexuality with older patients. It is very important for nurses' age and experience to be recognised as a barrier to meaningful communication with patients, as this can result in low quality of care and unmet patients' needs (Fennell and Grant 2019). Especially, with sexuality being a very difficult discussion topic, due to cultural, religious and personal taboos, the nurse/healthcare professional is expected to facilitate this discussion to all their patients, consistently (Chen, Jones, and Bannatyne 2020). Also, the two studies in which this theme has been revealed consisted of studies from the Western and Eastern cultures almost equally in numbers, placing this discussion on sexuality as a universal need, equally sought after by all prostate patients.

### 3.3 Personal Values and Attitudes

Social and personal factors can influence nurses' attitudes on sexuality. An astonishing 100% of the studies agree that nurses' personal values and attitudes have a significant impact on discussing sexuality with patients (Aling et al. 2021; Bauer, Haesler, and Fetherstonhaugh 2016; Chen, Jones, and Bannatyne 2020; Fabrício et al. 2021; Fennell and Grant 2019; Haesler, Bauer, and Fetherstonehaugh 2016; O'Connor, Connaghan, et al. 2019; Papadopoulou et al. 2019; Reese et al. 2017; Vassao et al. 2018; Williams and Addis 2021). The variance of attitudes and discomfort of the topic can account for why nurses neglect to discuss sexuality with patients (Fabrício et al. 2021). Embarrassment, lack of confidence and shame are the key emotions that deter nurses from initiating discussion on sexuality with patients (Bauer, Haesler, and Fetherstonhaugh 2016; Fennell and Grant 2019; O'Connor, Connaghan, et al. 2019; Papadopoulou et al. 2019; Vassao et al. 2018; Williams and Addis 2021).

More than one‐third (36%) of studies identified patients' older age as being a significant barrier to discussing sexuality with them (Bauer, Haesler, and Fetherstonhaugh 2016; Haesler, Bauer, and Fetherstonehaugh 2016; O'Connor, Connaghan, et al. 2019; Williams and Addis 2021). Haesler, Bauer, and Fetherstonehaugh (2016) illustrated that some nurses have the ageist view that sexuality is not important in the older population and that older people do not wish to discuss the topic.

Narrowing the focus to erectile dysfunction alone, however, obscures numerous other aspects of sexuality (Kokay, Power, and McGrath 2023). The misconception that medical interventions are the sole solution to health issues and no further discussion is necessary complicates the discussion even further (Vassao et al. 2018). Some nurses prefer to deal with male patients, as their issues in being physical (such as sexual dysfunction and infertility) can be easily rectified with a medical remedy or prescription without engaging in further communication and engagement with the patient (Haesler, Bauer, and Fetherstonehaugh 2016).

### 3.4 Organisational Factors

Nurses and healthcare professionals do not work in isolation. Their organisations provide them the structures and resources for quality and efficient services. Even though organisations try to put efficient structures and resources in place, it has been observed in the literature that deviations can happen. Nearly half (45%) of the included studies identified organisational factors as barriers to discussing sexuality with patients (Aling et al. 2021; Fabrício et al. 2021; O'Connor, Connaghan, et al. 2019; Papadopoulou et al. 2019; Williams and Addis 2021). These include shortage in the time allocated to the needs of these patients, due to rising patient numbers (Fabrício et al. 2021; Williams and Addis 2021), lack of privacy within clinical environments (Aling et al. 2021; O'Connor, Connaghan, et al. 2019; Papadopoulou et al. 2019; Williams and Addis 2021) and staff shortages in clinical areas (Aling et al. 2021; O'Connor, Connaghan, et al. 2019).

Additionally, the infrastructure within the service is also culpable (Aling et al. 2021; Papadopoulou et al. 2019). This refers to the lack of clear referral pathways to sexual health specialists, including sex therapists, once an issue is identified (Papadopoulou et al. 2019).

These overlapping themes illustrate the barriers that contribute to nurses not discussing sexuality with patients with prostate cancer. They also give insight to the discrepancy that exists between a nurses' knowledge and their clinical practice, potentially leading to unmet patient needs and distress.

## 4 Discussion

Sexuality is widely recognised as a crucial aspect of holistic care by most healthcare professionals (Agochukwu‐Mmonu et al. 2021; Burbage‐Veith 2020; Greimel et al. 2018; Krouwel et al. 2015; Leonardi‐Warren et al. 2016; Ussher et al. 2013). There is evidence however, that suggests there is a discrepancy between health professionals' ideology and their clinical practice, as many of the included studies reveal many healthcare professionals do not engage in discussions about sexuality with their cancer patients (Bauer, Haesler, and Fetherstonhaugh 2016; Fabrício et al. 2021; Haesler, Bauer, and Fetherstonehaugh 2016; Papadopoulou et al. 2019; Reese et al. 2017). Analysis of themes identified in this study illustrate the factors that can strongly influence the healthcare practitioners' willingness to discuss sexuality with their cancer patients.

### 4.1 Lack of Training and Education

A significant issue identified across the literature is lack of training and is identified in more than half of the studied articles (Chen, Jones, and Bannatyne 2020; Fennell and Grant 2019; Williams and Addis 2021). Despite evidence that nurses appreciate the significant role that sexuality has in patient's quality of life (Krouwel et al. 2015; Leonardi‐Warren et al. 2016), many nurses report that they feel ill‐equipped to engage in the conversation (Maree and Fitch 2019; Oskay, Can, and Basgol 2014). Some nurses believe that it is the role of the oncologist to discuss sexuality (Depke and Onitilo 2015). This aligns with past research, which states that even though nurses appear to be willing to discuss the topic of sexuality, < 11% of them do so frequently; the remaining admit to a lack of knowledge/sufficient level of education or confidence in answering related questions adequately. Furthermore, some nurses believe that their nurse training did not adequately prepare them to discuss this topic with confidence (Saunamaki and Engstrom 2014). The lack of guidance and mentorship during the early years of clinical practice contributes to nurses feeling insufficiently educated to discuss sexuality with patients (Saunamaki and Engstrom 2014).

Nurses, however, have a personal responsibility to pursue professional education and development to ensure their scope of practice (Saunamaki and Engstrom 2014). It is incumbent for nurses to create a safe and supportive environment for patients who wish to discuss personal issues surrounding their sexual health (Mendes 2018). While undergraduate training may be insufficient for specialist clinical practice (Chen, Jones, and Bannatyne 2020), further education and training can help nurses feel more confident to initiate discussions on sexual health issues with prostate cancer patients (Maree and Fitch 2019; O'Connor, Connaghan, et al. 2019). This can also help in rising nurses' motivation in addressing the holistic care needs of patients (Norouzinia et al. 2016). Continued education will also contribute to the establishment of appropriate evidence‐based best practice guidelines that include sexual health in routine assessments (Bauer, Haesler, and Fetherstonhaugh 2016; Burbage‐Veith 2020; Chen, Chang, et al. 2020; Krouwel et al. 2015).

### 4.2 Age and Clinical Years of Experience

A common barrier highlighted throughout this study is the age and years of clinical experience of nurses (Chen, Jones, and Bannatyne 2020; Haesler, Bauer, and Fetherstonehaugh 2016; Reese et al. 2017). This finding is supported by results that show nurses aged over 30 years and those who have more than 10 years clinical experience identify as more comfortable to discussing sexuality with their cancer patients (Huang et al. 2013). Furthermore, Krouwel et al. (2015) found that nurses with higher academic qualifications, more clinical experience in oncology care and greater knowledge in sexual health feel more well‐versed in addressing sexual health difficulties with cancer patients (Krouwel et al. 2015). This issue highlights a significant gap in the provision of quality holistic care as men with prostate cancer can face long‐term challenges and side effects of treatment (Prashar, Schartau, and Murray 2022). A study in 2013 (Cockle‐Hearne et al. 2013) discovered that 81% of over 1000 men living with various stages of prostate cancer across Europe reported having unmet supportive care needs. This high presence of unmet needs highlights the urgent need for more comprehensive supportive care (Prashar, Schartau, and Murray 2022) and greater awareness of and holistic approach to supportive care (Cockle‐Hearne et al. 2013). To address this unmet need, healthcare's quality systems should assure same quality care, regardless of the practitioners' age and/or experience. Further exploration and action on factors that prohibit younger and less experienced practitioners from meeting these patients' needs is recommended. With this aim as a guide, prostate patients could experience holistic and quality care throughout their cancer journey with regard to their sexuality (Almont et al. 2019).

### 4.3 Personal Values and Attitudes

Nurses' personal values can act as barriers to initiating discussion on sexuality with cancer patients, as highlighted in 27% of articles in this study (Bauer, Haesler, and Fetherstonhaugh 2016; Fennell and Grant 2019; O'Connor, Drummond, et al. 2019). These attitudes can vary but often include the belief that sexual function is not a concern for older patients (Haesler, Bauer, and Fetherstonehaugh 2016; Krouwel et al. 2015; Papadopoulou et al. 2019; Ussher et al. 2013). Healthcare professionals, including nurses, fail to provide adequate sexual health care to older patients due to a discriminative opinion that some patients are too old to have issues with sexuality (Bauer et al. 2013). However, studies show that a significant number of older men remain sexually active (Cybulsky et al. 2018).

Embarrassment surrounding the topic of sexuality can also dissuade healthcare professionals from discussing sexual health and sexuality with patients (O'Connor, Connaghan, et al. 2019). Many healthcare professionals may feel uncomfortable or reluctant to initiate such discussions, believing that it will make patients feel uncomfortable (Bauer et al. 2013; Greimel et al. 2018; Leonardi‐Warren et al. 2016; O'Connor, Connaghan, et al. 2019). However, patients with prostate cancer often consider sexual dysfunction and changes to sexuality as being significant negative changes, affecting their quality of life (Eroglu and Ozkan 2023).

Practitioners' embarrassment often make them focus on patients' physical healthcare alone; this can hinder discussion on sexuality, as it may neglect the emotional and psychosocial aspects of sexual dysfunction experienced by patients (Haesler, Bauer, and Fetherstonehaugh 2016; Wasserug, Westle, and Dowsett 2017). Addressing erectile dysfunction solely through medication may not adequately address patients' broader needs. A more comprehensive approach that considers biological, psychological and social factors is recommended to address sexual dysfunction and sexuality in prostate cancer patients (Schover et al. 2014; Wasserug, Westle, and Dowsett 2017). For this reason, taking a more multidisciplinary approach, rather than relying solely on medication, can lead to more effective management strategies (Couper et al. 2015; King et al. 2015).

Also, the barrier of embarrassment is not limited to any specific gender or sexuality and is recognised across patient populations (Prashar, Schartau, and Murray 2022). Gay and bisexual men, in particular, report gaps in their psychosocial care, as healthcare professionals fail to address their concerns regarding the impact of cancer and treatment on their sexuality (Doran et al. 2018; Hoyt et al. 2017; McConkey and Holburn 2018). This emphasises the gaps for healthcare professionals to initiate discussion on sexual health and provide information and support to address patients' psychosocial needs (Prashar, Schartau, and Murray 2022). Cancer patients need and want healthcare professionals to initiate the discussion on sexual health and to provide information and advice (Komlenac and Hochleitner 2020; Sporn et al. 2015; Ussher et al. 2016).

### 4.4 Organisational Factors

Healthcare has been trying to cope with the burden of ageing population and nursing practitioners' shortage globally and cancer care is not immune to this reality. Staff shortage and lack of resources (time, clinical space, etc.) are organisational factors that affect clinical care. These organisational factors can affect sexual health and sexuality discussions as identified in 45% of the articles in this research (Fabrício et al. 2021; O'Connor, Connaghan, et al. 2019; Papadopoulou et al. 2019; Vassao et al. 2018; Williams and Addis 2021). The two most common organisational factors that contribute to barriers are shortage of time to spend with prostate patients and staff shortages, which result in prostate cancer patients not receiving adequate attention to their sexual health (Fabrício et al. 2021; Greimel et al. 2018). Another key consideration is the lack of a congruent pathway for referring patients to appropriate specialists and experts such as sex therapists (Oskay, Can, and Basgol 2014). Research carried out by Oskay, Can, and Basgol (2014) identified that 69% of the nurses who participated reported that there was no referral pathway to professional experts for patients who have a problem with sexuality. This finding showed that not having experts in sexuality within the multidisciplinary team can be a barrier to the topic being discussed with patients.

These challenges highlight the need for a comprehensive and multidisciplinary approach. Teamwork, especially within multidisciplinary groups, is challenging, and effective communication and collaboration among healthcare professionals and experts is crucial. Being successful in this could lead to the development of support groups, patient information literature and referral pathways to address these barriers (Li, Gao, and Wang 2016). By working together and prioritising patients' wellbeing, healthcare can address these organisational challenges and provide comprehensive support for prostate cancer patients regarding their sexuality.

## 5 Conclusion

Prostate cancer and its treatments can have an adverse effect on a patient's sexuality and masculine identity. The illness and its treatments can result in a negative impact on body image, sexual function and masculinity. Providing care to patients with prostate cancer requires expertise in communication, knowledge and sensitivity. Nurses are pivotal in providing a high standard of quality care that is individualised to their patients (Moore, Higgins, and Sharek 2013). Yet it remains clear that barriers exist and hinder effective discussion on sexuality with prostate cancer patients. Organisational factors such as time constraints within clinical practice, staff shortages and lack of referral paths have been identified as the most common barriers to addressing this topic. Establishing a comprehensive referral system of experts in the field of sexual health is also essential. This ensures that specialised expertise and supports are readily available to patients when needed. Just as any other treatment, side effects are addressed through a multidisciplinary team approach; the topic of sexuality should be treated in a similar matter. A collaborative approach actively involving various healthcare professionals can create a supportive environment for all.

Next, a lack of relevant education and training, age, clinical years of experience and personal values and attitudes were factors that also hindered the much‐needed initiation of discussion on sexuality with cancer patients. Education is crucial to equip nurses with the necessary skills and knowledge to overcome personal discomfort and address the topic of sexuality confidently. Integrating mandatory education on sexuality in specialised nursing programs will help nurses navigate any taboos or discomfort associated with this subject. This, in turn, can encourage nurses to initiate conversations on sexuality with patients fostering an environment of open communication.

By addressing the barriers, providing education and establishing a collaborative approach, nurses can contribute to a higher standard of individualised care for patients with prostate cancer.

### 5.1 Strengths and Limitations

A thorough review procedure and systematic approach was applied in this umbrella review, and it provides a strong foundation for the findings and conclusions. The inclusion of systematic review papers from various geographical regions allows for a multicultural viewpoint, which is important for our diverse nursing and patient population.

However, the underrepresentation of men from different sexual groups, including those from the LGBTIQ community, suggests potential gaps in addressing their specific needs. Further research is advised.

## Author Contributions

**Catherine Neenan:** conceptualisation, data curation, formal analysis, investigation, methodology, project administration, resources, validation, visualisation, writing – original draft and writing – review and editing. **Anna V. Chatzi:** conceptualisation, data curation, formal analysis, investigation, methodology, project administration, validation, visualisation and writing – review and editing.

## Conflicts of Interest

The authors declare no conflicts of interest.

## Peer Review

The peer review history for this article is available at https://www.webofscience.com/api/gateway/wos/peer‐review/10.1111/jan.16703.

## Data Availability

Data available upon request.
